# Towards condom skills: a cross-sectional study of the association between condom proficiency, condom problems and STI risk amongst MSM

**DOI:** 10.1186/1471-2458-12-747

**Published:** 2012-09-06

**Authors:** Lisa Goodall, Daniel Clutterbuck, Paul Flowers

**Affiliations:** 1 Chalmers Centre, 2A Chalmers Street, Edinburgh EH3 9ES, UK; 2 School of Health and Life Sciences, Glasgow Caledonian University, Glasgow G4 0BA, UK

**Keywords:** Condom skills, Men who have sex with men, Sexually transmitted infections, Condom problems, Condom proficiency, Condom measures

## Abstract

**Background:**

Condom use problems are common amongst Scotland’s men who have sex with men (MSM). To date condom errors have been associated with the likelihood of sexually transmitted infections in heterosexual sexually transmitted infection (STI) clinic attendees but not in MSM and direct evidence of a link between condom problems and STI acquisition in MSM have been lacking. This study investigated the possibility of an independent association between condom proficiency, condom problems and STI acquisition in MSM in Scotland.

**Methods:**

An exploratory observational design employed cross-sectional surveys in both STI clinic and community settings. Respondents completed self-report measures of socio-demographic variables, scales of condom proficiency and condom problems and numbers of different partners with whom men have had unprotected anal intercourse (UAI partners) in the preceding year. Self-report data was corroborated with clinical STI diagnosis where possible. Analysis included chi-squared and Mann–Whitney tests and multiple logistic regression.

**Results:**

792 respondents provided data with an overall response rate of 70% (n = 459 clinic sample, n = 333 community sample). Number of UAI partners was the strongest predictor of self-reported STI acquisition over the previous 12 months (p < 0.001 in both clinic and community samples). Demographic characteristics were not associated with self-reported STI diagnosis. However, condom proficiency score was associated with self-reported STI acquisition (p < 0.05 in both samples). Condom problem score was also associated with self-reported STI diagnosis in the clinic (p = 0.001) but not the community sample. Condom problem score remained associated with self-reported STI diagnosis in the clinic sample after adjusting for number of UAI partners with logistic regression.

**Conclusions:**

This exploratory study highlights the potential importance of targeted condom use skills interventions amongst MSM. It demands further research examining the utility of condom problem measures in wider populations, across prospective and experimental research designs, and a programme of research exploring their feasibility as a tool determining candidacy for brief interventions.

## Background

In common with findings in some other countries [1], cross sectional surveys amongst bar–going gay men in Scotland showed an increase in rates of unprotected anal intercourse (UAI) in the late 1990s [2] followed by a plateau [3], with men under 25 years of age consistently showing higher rates of UAI than older men. Whilst prevention is usually thought of as encouraging men to use condoms *per se*, here the issue of the effective use of condoms is examined.

Behavioral interventions continue be an essential component of HIV prevention in MSM [4] but targeting evidence based interventions to achieve maximum benefit is challenging. MSM attending clinics exhibit variable levels of sexual risk behavior and the differences in risk behavior, when assessed through routine clinical questioning, between men testing HIV + ve and HIV-ve are small [5]. Clinic and community based behavior change interventions that reduce UAI and partner number in MSM are resource intensive [6] and the criteria for the selection of men who will benefit most from such interventions are to date unclear. Reducing onwards transmission by improving condom use amongst those who use them presents an underexplored area of sexual health research. Moreover, increasing condom skills may well reduce negative condom experiences and lead to an overall increase in condom use.

The effectiveness of condoms in preventing sexually transmitted infections (STI) including HIV depends on consistent and correct use [7]. The frequency and correlates of condom use problems and errors have been reported in a range of populations [8] and in men engaging in vaginal sex and anal sex with female partners [9] and anal sex with male partners [10]. Recent research also indicates that condom use problems are common among Scotland’s MSM. A 2008 cross-sectional study of MSM recruited from the gay bars of Glasgow and Aberdeen, or men receiving condoms through a Scottish postal distribution scheme, found that 27% of men reported condom breakage and 40% condom slippage in the last year [11]. To date condom errors have been associated with the likelihood of sexually transmitted infections in heterosexual STI clinic attendees [12] but not in MSM [13] and direct evidence of a link between condom problems and STI acquisition in MSM has so far been lacking.

This exploratory study addresses this lacuna. It investigates the associations between demographics, STI risk, condom proficiency and condom errors in MSM and the likelihood of STI acquisition among MSM. It has been designed to inform both the selection of candidates for brief behavior change interventions in clinic and community settings, and to guide the design and content of such interventions.

## Methods

A cross-sectional questionnaire study of MSM attending STI clinics and community settings; including gay-identified bars, clubs and saunas was performed over a 16-week period ending 27 August 2010. The self-completed, pen-and-paper, anonymous questionnaire was based on a framework that had previously been successfully used in the triennial MRC bar-based Gay Men’s Sexual Health Surveys to assess HIV prevalence and sexual attitudes and behaviors in Scottish MSM [2-4]. The questionnaire was further developed in consultation with sexual health clinicians and MSM representatives who advised on question style and content.

Pilot questionnaires were trialed on 15 clinic attendees to assess suitability of questionnaire length and to identify potential difficulties with comprehension or presentation of questions within this particular population. The pilot study indicated clinic attendees were happy to participate and most were able to fully complete the questionnaire within 10 minutes. Following the pilot, minimal adjustments were made to the wording of some questions in order to increase clarity. The questionnaire recorded data on demographics, sexual activity, condom use for anal sex and condom problems and assessed condom knowledge and proficiency. Self-reported data on STIs diagnosed in the last 12 months were also collected. For the purpose of this study ‘STIs’ included gonorrhea, syphilis, chlamydia, HIV, Hepatitis B and Hepatitis C only; diagnoses of genital warts and herpes simplex virus were excluded due to difficulty distinguishing between incident and recurrent infections.

In clinic attendees additional consent was requested to allow the questionnaire to be linked anonymously to laboratory STI results by attaching a unique patient identifier to each questionnaire and to laboratory request forms. The unique identifier was used to obtain results of STI tests from each participant at that clinic visit. This enabled the matching of self-report data with clinical data for a subset of the sample (n = 333), while avoiding the exclusion of men who were not happy for their clinical records to be linked to the questionnaire.

Information leaflets describing the study were available in both clinic and community settings. In clinics, questionnaires were distributed by receptionists, nurses, health advisors and doctors to all men identified as MSM attending the clinic during the study period. In community settings an established time and location sampling methodology [14] was used to recruit a representative sample of men visiting the gay bars and saunas of Edinburgh (6 different settings). Trained volunteers distributed all questionnaires in community settings, offering questionnaires to all men attending each venue during the sampling period. Response rates were recorded for both clinic and community arms of the study.

### Study participants

All MSM aged over 16 years attending STI clinics or community venues were eligible to take part in the study. The survey was only available in English language. However, interpreters were offered when required to enable men whose first language was not English to participate. An interpreter was needed by only one Polish male who participated in the clinic arm of the study.

### Ethical approval

Ethical approval for this study was granted by the Lothian Research Ethics Committee.

### Statistical methodology

A power calculation prior to commencing the study suggested that a sample size of 500 would allow estimation of rates of condom use, knowledge and errors to within a standard error of not more than 2%. It would give 80% power to detect as significant differences of the order of 13% in the rates of condom variables between approximately equal-sized groups based on demography. Review of STI rates in MSM attending clinics over the previous 2 years showed that these were unlikely to exceed 15% even for all infections combined, so power would be considerably lower for detecting associations between condom use and incident rates of STI. Major reductions of the order of 40% in STI rates in those using condoms relative to those not doing so would be detectable with high power. An additional measure of self-reported STI was included to ensure that STI data was available on both clinic and community samples and to increase the probability of detecting associations between demographic and behavioral variables and STI acquisition.

Data were analysed using SPSS version 18.0. Univariate associations between independent variables and STI diagnosis were tested by chi-squared tests for categorical variables and Mann–Whitney tests for continuous variables. Multiple logistic regression was used to control for the effects of multiple factors on STI diagnosis. Logistic regressions were conducted separately for clinic and community samples since factors significantly associated with STI diagnosis differed between these two samples. Factor analysis was carried out by principal component analysis.

### Condom proficiency and condom problem scales

Questions on condom proficiency, knowledge and problems were developed from those showing the most frequent positive responses in published studies [7,13], those reported by Scottish MSM [11] and questions used in one or more validated MRC bar survey tools [3,4]_._ New questions were tested for face validity and revised. Responses were combined and tested for reliability using factor analysis to produce a scale of condom proficiency and a scale of condom problems.

*The condom proficiency scale* combined responses to 3 questions on ease of having safer sex, confidence in using condoms and ease of negotiating condom use with partners for anal sex. The answers were all on 4-point ordinal scales (Table 1). In a factor analysis, the first principal component had approximately equal coefficients for each of the questions, suggesting that the sum of these would be a suitable composite scale with a range of 0–9 and a mean of 1.42 for the 757 cases for which all 3 items were scored by respondents. Cronbach’s alpha for this scale was 0.71, indicating acceptable internal consistency (Cronbach’s alpha scores greater than 0.5 may indicate some degree of internal consistency with higher scores, closer to 1, indicating greater internal consistency [15]).

**Table 1:** Questions combined to assess condom proficiency

**Question**	**Possible responses**
How easy is it for you to have safer sex? (tick one)	· Very easy
	· Quite easy
	· Quite difficult
	· Very difficult
How confident are you at using condoms? (tick one)	· Not at all confident
	· Slightly confident
	· Confident
	· Very confident
How easy is it for you to negotiate condom use with partners? (tick one)	· Very easy
	· Quite easy
	· Quite difficult
	· Very difficult

*The condom problem scale* was a cumulative score created by adding the number of condom problems reported by individuals in response to questions on having difficulty finding condoms that fit and experience of problems with condom use, both over the last 12 months and at last sexual encounter (Table 2). A higher value was associated with a higher condom problem score. This scale had a total of 23 ‘yes/no’ responses. Factor analysis again suggested that the sum of these would be a suitable composite scale with a range of 0–23 and a mean of 1.47 for the 764 cases in which all questions were answered by respondents. Cronbach’s alpha for the condom problem scale was 0.66.

**Table 2:** Questions combined to assess condom problems

**Question**	**Possible responses**
Do you have problems obtaining condoms that fit your penis? (tick one)	· Yes
	· No
If you used condoms for anal sex in the last 12 months were there any problems with the condom? (tick as many as apply)	· Condom defective when removed from packet
	· Started to put the condom on inside-out
	· Lost erection before putting condom on penis
	· Tore condom with sharp object before use
	· Had allergic reaction or irritation from condom
	· Condom split/burst
	· Condom slipped off
	· Condom was removed during sex
The following can increase the chance of a condom tearing or slipping off. In the last year which of these have you done? (tick as many as apply)	· Used a condom without waterbased lubricant
	· Used saliva as lubricant
	· Fully unrolled the condom before putting on
	· Used a condom that was too small for you
	· Put lots of lubricant inside the condom before putting it on
If you used a condom last time you had anal sex were there any problems with the condom? (tick as many as apply)	· Condom defective when removed from packet
	· Started to put the condom on inside-out
	· Lost erection before putting condom on penis
	· Tore condom with sharp object before use
	· Had allergic reaction or irritation from condom
	· Condom split/burst
	· Condom slipped off
	· Condom was removed during sex
If you used a condom last time you had anal sex was the condom put on before penetration? (tick one)	· Yes
	· No

## Results

459 clinic and 333 community questionnaires were returned. Response rates were 70% in both clinic and community samples. 666 MSM attended clinic during the study period, 268 of whom were known to be HIV positive (40%). 98% (652) of all MSM attending clinic during the study period were offered questionnaires. 22.2% of men in the clinic sample and 15.1% of men in the community sample reported being diagnosed with an STI over the previous 12 months. In the clinic sample, 333 men consented to questionnaire linkage with STI results. 42 STIs were diagnosed in 38 men.

The mean age of men was 36 (range 17–74) in the clinic sample and 38 in the community sample (range 17–73). The majority of participants in both samples were of white UK ethnicity; 79% of clinic and 81% of community sample; and most were employed; 72% of clinic and 76% of community sample. Men in both samples tended to be highly educated; 58% of the clinic and 55% of the community sample were educated to undergraduate degree level or above. 36% of the clinic sample and 7% of the community sample self-reported as being HIV positive. None of these demographic characteristics were associated with self-reported STI acquisition over the previous 12 months (Table 3).

**Table 3:** Tests of association between independent variables and self-reported STIs over the previous 12 months

	**Clinic (% of n)**	**Self-reported STIs (% of category)**	**P**	**Community (% of n)**	**Self-reported STIs (% of category)**	**p**
**Age**	n = 445	n = 97		n = 323	n = 46	
<30	154 (34.6)	33 (21.4)	0.95	104 (32.2)	15 (14.4)	0.26
30–39	128 (28.8)	29 (22.7)		69 (21.4)	14 (20.3)	
40+	163 (36.6)	35 (21.5)		150 (46.4)	17 (11.3)	
**Educational qualifications**	n = 418	n = 91		n = 288	n = 39	
None	8 (1.9)	4 (50.0)	0.15	10 (3.5)	1 (10.0)	0.55
GCSEs/O-levels/Standards	72 (17.2)	14 (19.4)		37 (12.8)	9 (24.3)	
A-levels/Highers	65 (15.6)	12 (18.5)		53 (18.4)	7 (13.2)	
Vocational	8 (1.9)	3 (37.5)		11 (3.8)	1(9.1)	
HNC or HND	24 (5.7)	9 (37.5)		18 (6.3)	3 (16.7)	
Undergraduate degree	194 (46.4)	39 (20.1)		130 (45.1)	14 (10.8)	
Postgraduate degree	47 (11.2)	10 (21.3)		29 (10.1)	4 (13.8)	
**Ethnicity**	n = 450	n = 100		n = 332	n = 50	
White (UK)	357 (79.3)	80 (22.4)	0.73	257 (80.6)	32 (12.5)	0.06
White (Irish)	16 (3.6)	3 (18.8)		19 (6.0)	4 (21.1)	
White (other)	45 (10.0)	8 (17.8)		34 (10.7)	7 (20.6)	
All non-white	32 (7.1)	9 (28.1)		22 (0)	7 (31.8)	
**Employment status**	n = 444	n = 97		n = 324	n = 46	
Employed	283 (63.7)	63 (22.3)	0.08	220 (67.9)	35 (15.9)	0.34
Self-employed	38 (8.6)	10 (26.3)		26 (8.0)	3(11.5)	
Unemployed	50 (11.3)	13 (26.0)		35 (10.8)	4 (11.4)	
Student	61(13.7)	9 (14.8)		15 (4.6)	2 (13.3)	
Retired	12 (2.7)	2 (16.7)		28 (8.6)	2 (7.1)	
**Self-reported HIV status**	n = 385	n = 89	0.11	n = 253	n = 23	0.93
HIV negative	246 (63.9)	56 (22.8)		235 (92.9)	19 (8.1)	
HIV positive	139 (36.1)	33 (23.7)		18 (7.1)	4 (22.2)	
**Number of UAI partners**	n = 445	n = 97		n = 296	n = 31	
0	219 (49.2)	30 (13.7)	**<0.001**	172 (58.1)	12 (7.0)	**<0.001**
1	129 (29.0)	28 (21.7)		80 (27.0)	7 (8.8)	
2–4	78 (17.5)	27 (34.6)		25 (8.4)	9 (36.0)	
5–10	10 (2.2)	6 (60.0)		7 (2.4)	1 (14.3)	
>10	9 (2.0)	6 (66.7)		12 (4.1)	2 (16.7)	

Number of UAI partners over the previous year was used as a measure of men’s STI risk. The mean number of UAI partners in the previous 12 months was 1.5 in the clinic sample and 3.3 in the community sample. The range of UAI partners was wide in both samples (range 0–100 in the clinic sample and 0–245 in the community sample). In order to test associations between number of UAI partners and STI diagnosis, and to account for the possible influence of the few men reporting very high numbers of UAI partners, number of UAI partners was categorised as 0, 1, 2–4, 5–10, or >10. Number of UAI partners was found to be the strongest predictor of self-reported STI acquisition over the previous 12 months (Table 3).

The condom proficiency score was significantly associated with self-reported STI diagnosis over the previous 12 months in both clinic and community samples. The condom problem score was associated with self-reported STI diagnosis in the clinic but not the community sample (Table 4). Number of UAI partners (i.e. neither condom proficiency nor problem score) was the only variable to be significantly associated with laboratory confirmed STIs among the clinic sample.

**Table 4:** Tests of association between condom proficiency and condom problem scores and self-reported STIs over the previous 12 months

**Source**	**Self-reported STI**		**Condom proficiency score**	**p**	**Condom problem score**	**p**
Community	Yes	Mean	1.93	**0.012**	1.33	0.757
		N	44		45	
		Standard deviation	2.07		1.82	
	No	Mean	1.29		1.54	
		N	267		274	
		Standard deviation	1.84		2.40	
	Total	Mean	1.38		1.51	
		N	311		319	
		Standard deviation	1.88		2.33	
Clinic	Yes	Mean	1.77	**0.032**	1.97	**0.001**
		N	99		97	
		Standard deviation	1.68		1.95	
	No	Mean	1.36		1.26	
		N	347		348	
		Standard deviation	1.43		1.54	
	Total	Mean	1.45		1.41	
		N	446		445	
		Standard deviation	1.50		1.67	

All variables found to be associated with self-reported STI diagnosis in univariate analysis (number of UAI partners, condom proficiency score and condom problem score) were entered into a logistic regression model. Number of UAI partners in both samples and condom problem score among the clinic sample remained significant (Table 5) in explaining the variance in self-reported STI diagnosis. That is, among the clinic sample, condom problem score remained significantly associated with self-reported STI diagnosis after adjusting for UAI risk. In the community sample condom problem and proficiency scores were not associated with self-reported STI diagnosis after adjusting for UAI risk.

**Table 5:** Logistic regressions illustrating contribution of multiple variables in predicting the likelihood of self-reported STI diagnosis

**Sample**	**Variable**	**Estimate**	**95% CI**	**p**
Clinic	Condom problem score	−0.154	−2.178 – −1.493	**0.029**
	Number UAI partners	−1.073	−1.602 – −0.543	**<0.001**
Community	Condom problem score	0.049	−0.133 – 0.232	0.596
	Number UAI partners	−1.608	−2.457 – −0.758	**<0.001**
Clinic	Condom proficiency score	−0.113	−0.264 – 0.038	0.144
	Number UAI partners	−1.133	−1.645 – −0.621	**<0.001**
Community	Condom proficiency score	0.114	−0.150 – 0.295	0.524
	Number UAI partners	0.484	−2.613 – −0.715	**0.001**

## Discussion

For the first time in the UK, this study found significant associations linking measures of condom problems to STI risk amongst MSM. Although condom errors and other problems have been increasingly recognised as a likely factor in reducing condom effectiveness [16] and the correlates of condom problems in men have been explored [7,17], biological end point evidence of an association with STI acquisition is scarce in all groups and absent in MSM. An association between condom problems and incident chlamydial and gonococcal infections was shown in a small subsample (21 infections in 130 individuals) of the large Project Respect intervention trial [18]. In a study of 1973 consistent condom users, condom errors were reported much less frequently by MSM than men who have sex with women and an association between condom problems and prevalent STI was seen in heterosexual but not homosexual subjects [13]. We found strong univariate associations between condom proficiency and problem scores and measures of STI acquisition, although only condom problem score remained independently associated after correction for UAI partners. It is entirely conceivable that men who report lower condom proficiency and more condom problems are less likely to use condoms and this requires further exploration. Critically, our study found independent effects of condom problems in relation to self-reported STI acquisition and not laboratory confirmed STIs among the clinic sample. This too is suggestive of a link between condom problems and other risk behaviors rather than a direct link with STI transmission. However, it is important to note that the sample size for men who were found to have a laboratory confirmed STI was small (n = 38); it is therefore possible that sample size may have meant inadequate power to detect any association between condom problems and laboratory confirmed STIs that did exist.

Number of UAI partners was strongly associated with self-reported STI diagnosis over the previous 12 months as well as with prevalent STI. This is unsurprising and is consistent with other behavioral community based surveys of self-reported STIs in MSM [19] and with clinic based surveys of HIV positive MSM [20]. With evidence of ongoing high-risk behavior in some MSM there is clearly a continuing need for interventions to promote condom use to successfully reduce STI acquisition.

Men at high risk of HIV in Scotland are in contact with prevention services [21] and interventions are likely to be acceptable [22]. A UK study to determine whether condom thickness affects condom breakage found that user characteristics, including lack of confidence in condom use, rather than condom characteristics were associated with condom breakage among MSM [23]. Although clinic based behavioral interventions have been proven to reduce the risk of STIs in a large randomized controlled trial [24] it is recognised that these interventions are resource intensive and recent work has focused on reducing the duration and frequency of interventions. Recent studies have shown a reduction in repeat STIs following a single session of condom skills training delivered to young heterosexual African American men by a lay health adviser [25]. A small pilot study of home based condom skills training showed some improvement in measures of condom self-efficacy and reduction in condom errors in 30 young heterosexual men [26]. The condom self-efficacy and error scores used in that study had high internal consistency but have not been shown to be indicators of STI risk. There is good evidence that intensive multi-session clinic based brief behavior change interventions with MSM can reduce the frequency of UAI with partners of unknown HIV status [27] but there is a lack of evidence on less intensive single session interventions or interventions designed to reduce condom errors in MSM. It is possible that the lower frequency of condom problems in MSM might reduce the impact of condom skills training – although interventions to reduce problems may increase overall levels of use. Such interventions could focus on a number of condom skills, for example, appropriate sized condoms, skills relating to appropriate use or skills relating to condom communication and negotiation.

Strengths of our study included use of both clinical and community samples, the high proportion of HIV positive men included in the sample and the matching of self-report risk behavior to clinical records. Limitations of the study include convenience sampling of populations at selected venues. This could limit the transferability of study findings to MSM populations in other settings and locations. Epidemiological studies show that MSM recruited from community settings have higher rates of undiagnosed HIV infection than those recruited from STI clinics [14] and men in different community samples report widely differing rates of UAI [28,29]. The effect of convenience sampling was mitigated by recruiting from both STI clinics and community venues. All ‘gay identified’ commercial venues in Edinburgh were included. All MSM attending STI clinics and all men attending each community venue during the sampling period; which included different weekdays and different sample times over the 4-month study period; were asked to participate in an attempt to obtain a representative sample. Response rates were high for both clinic and community samples although it is acknowledged that high response rates do not necessarily mean a representative samples. As the study was designed to inform the development of prevention interventions which are likely to be delivered through the same clinics and commercial venues and to the same population, this methodology also carries some advantages. A further limitation is the reliance of this study on subjective reporting of condom use, condom problems, sexual behavior and STI diagnoses. This may have resulted in misclassification of both exposure and outcome. MSM participating in the study may have under-reported high-risk sexual behavior in an attempt to satisfy perceived social norms, but the reported levels of UAI suggest that this occurred to no greater extent than in other published studies and surveys. The use of self-completed anonymous questionnaires may have reduced under-reporting by maintaining anonymity, although further improvements may have been seen through the use of computer–assisted survey technology. Any under-reporting of high-risk sexual behavior is likely to have been evenly distributed throughout the samples, resulting in non-differential bias. This would have lead to an under-estimation of any association between risk behavior and STI diagnosis and is unlikely therefore to change any of our conclusions. It was also not possible to estimate the incidence of STIs using our cross-sectional methodology or to establish any temporal relationship. Another limitation is the moderate internal consistency within the condom problem score. The condom problem score included a large number (23) of relatively varied and potentially unrelated issues, which may happen in isolation or in different circumstances. This limits its value as a global measure and suggests that a refinement of the score, informed by other recent publications [8-10] might be possible.

Despite these limitations, the study findings did allow hypotheses to be formed around factors that may be associated with STI diagnosis and the types of intervention that may be successful, highlighting possible areas for further study. There is good evidence that condom problems were more important than demographic characteristics, including HIV status, in explaining the variance of STI acquisition among the clinic sample of MSM. While causality cannot be inferred from this study, the study adds to the evidence base in providing a link between scores of condom problems and biological endpoints. Moreover it directs research attention to focus upon the feasibility and development of condom skills interventions in this population. A refinement of the condom problem scale devised in this study, or modifications of the scale could potentially be used as a measure of the effectiveness of behavioral interventions and might also allow larger scale observational studies with the power to confirm or refute associations between reported condom problems and incident STI. Single items or subsets of items from the scale also have potential for use as a tool for the clinical targeting of behavioral interventions in MSM. Moreover, targeting those who experience condom problems may improve the overall frequency and consistency of condom use, regardless of whether condom problems themselves are causal in the acquisition of STI in MSM.

## Conclusions

This exploratory study highlighted the potential importance of targeted condom use skills interventions amongst MSM. The study also emphasised the need for further research to examine the utility of condom problem measures in targeting behavioral interventions to improve sexual health amongst MSM and to explore the link between condom problems and the desired biological endpoint, a reduction in STI acquisition.

## Abbreviations

MSM: Men who have sex with men; STI: Sexually transmitted infections; UAI: Unprotected anal intercourse; UAI partners: Partners with whom men have had unprotected anal intercourse.

## Competing interests

The authors declare they have no competing interests.

## Authors’ contributions

DC and PF initiated the project. LG collected patient data. All authors contributed to the preparation of the manuscript.

## Pre-publication history

The pre-publication history for this paper can be accessed here:

http://www.biomedcentral.com/1471-2458/12/747/prepub
